# Low Incidence of Venous Thromboembolism and Pre-Eclampsia in Women Cared for in a Specialist Antenatal Clinic for Inflammatory Bowel Disease

**DOI:** 10.3390/jcm14228072

**Published:** 2025-11-14

**Authors:** Amy Gosling, Sofia Rellou, Konstantina Rosiou, Tracey Glanville, Christian Philipp Selinger

**Affiliations:** 1 Leeds Gastroenterology Institute, Leeds Teaching Hospitals NHS Trust, St James University Hospital, Bexley Wing, Leeds LS9 7TF, UK; amy.gosling3@nhs.net (A.G.); sofrellou@gmail.com (S.R.); konstantina.rosiou@nhs.net (K.R.); 2 Department of Gastroenterology, General Oncology Hospital of Kifisia, Timiou Stavrou 14, Kifisia, 145 64 Athens, Greece; 3 Department of Obstetrics, Leeds Teaching Hospitals NHS Trust, Leeds LS9 7TF, UK; tracey.glanville@nhs.net

**Keywords:** inflammatory bowel disease, pregnancy, venous thromboembolism, pre-eclampsia

## Abstract

**Background/Objectives**: Pregnant women with Inflammatory Bowel Disease (IBD) require careful management to ensure optimal maternal and fetal outcomes. Whether IBD is a risk factor for pre-eclampsia is controversial. We aimed to investigate the incidence of venous thromboembolism (VTE), pre-eclampsia, and risk of disease flare from low-dose aspirin. **Methods:** This is a retrospective study of the Leeds combined IBD antenatal clinic providing joint specialist gastroenterology and obstetric consultations from 2015 to 2024. Primary outcomes were incidence of VTE, incidence of pre-eclampsia, and disease flare whilst taking aspirin. **Results:** In 574 pregnancies a single episode of VTE occurred. Adherence to VTE prophylaxis guidelines was good for post-partum periods but only 2 of 51 cases with third-trimester flare received the required VTE prophylaxis. The incidence of pre-eclampsia in this cohort was low at 2.7%. The majority of women deemed high-risk for pre-eclampsia received prophylaxis with aspirin in accordance with guidelines. The risk of symptomatic disease flare was not increased for aspirin users (21.6% versus 22.2% for non-users (*p* = 0.61)). **Conclusions:** The incidence of VTE and pre-eclampsia was low in this cohort of pregnant women with IBD. Pre-eclampsia prophylaxis in adherence to guidelines was good and was not associated with an increased flare risk. VTE prophylaxis for third-trimester flares was insufficient and requires better attention from IBD specialists, who are most likely to encounter patients with IBD flares.

## 1. Introduction

Pregnant women with Inflammatory Bowel Disease (IBD) require careful management to ensure optimal maternal and fetal outcomes [1]. Active disease should be treated to avoid poor maternal outcomes and associated risks of low gestational weight for age, premature labour, and placental disease [2]. When care is provided in specialist settings most women with IBD experience good maternal and fetal outcomes [3,4]. Care is however delivered frequently in non-specialist settings and communication between IBD and obstetric teams is often ad hoc, which impacts true muti-disciplinary care [5].

Guidelines for the management of IBD during pregnancy focus mostly on the direct implications of IBD on maternal and fetal health [1,6]. The risk for maternal diseases of pregnancy is often managed by the obstetric team alone and may not be part of routine considerations for IBD clinicians. Venous thromboembolism (VTE) and pre-eclampsia pose serious risk to maternal health and can prove fatal if left undetected and untreated [7,8]. Safe and effective prophylaxis is available with low-molecular-weight heparin for VTE and low-dose aspirin for pre-eclampsia [7,8].

The main challenge for clinicians is to identify patients at risk for VTE and pre-eclampsia. Data for VTE show a clear risk increase in women with IBD that is mainly related to active IBD during the third trimester [9,10]. In contrast, data for pre-eclampsia are controversial, with studies showing no general increased risk of pre-eclampsia in all patients with IBD, but one study showed an increased risk for severe pre-eclampsia in IBD only [11]. Subsequently a discrepancy in guidelines arose with recent global guidelines recommending aspirin prophylaxis for all pregnant patients with IBD, while UK and European guidelines do not recommend this routinely in the absence of other risk factors for pre-eclampsia [1,8,12].

Prophylaxis for VTE with low-molecular-weight heparin is not controversial in non-pregnant patients [13]. In contrast, some clinicians and patients are concerned about the use of aspirin in IBD. While non-steroidal anti-inflammatory drugs are generally avoided in IBD due to a potential risk of increased flares [14], data on aspirin use in all IBD patients are scarce with a single reassuring study [15].

We aimed to investigate the incidence of venous thromboembolism (VTE), pre-eclampsia, and risk of disease flare from low-dose aspirin in the setting of a specialist combined IBD antenatal clinic.

## 2. Methods

### 2.1. Study Design and Setting

The Leeds combined IBD antenatal clinic provides joint specialist gastroenterology and obstetric consultations [4]. This study is a retrospective cohort study of women who attended the antenatal IBD clinic and subsequently delivered at Leeds Teaching Hospitals Trust between 2015 and 2024. We included all women with a known pregnancy outcome after week 20 including terminations, still birth and live birth. Patients who suffered a miscarriage before 20 weeks of gestation were excluded as the outcomes of interest mainly apply to pregnancy of weeks 20 gestation or more. Women who delivered elsewhere (pregnancy outcome data not available) or attended for pre-pregnancy counselling only were excluded from analysis.

Patient care for IBD was based on the European Crohn’s and Colitis Organisation guidelines [1,16], while risk assessments and prophylactic treatments for VTE were based on Royal College of Obstetricians and Gynaecologists guidelines [7]. Risk assessments and prophylactic treatments for pre-eclampsia were based on algorithms from Saving Babies Lives: Version 3 and National Institute for Health and Care Excellence guidance [8,17]. VTE prophylaxis was given in the form of weight-based dosing of low-molecular-weight heparin commencing at gestational week 28 for active IBD or earlier if additional risk factors were present according to the risk assessment. Cessation of low-molecular-weight heparin was determined on the actual risk calculated for each patient and was continued for 6 weeks in high-risk women and for 10 days in intermediate-risk women. Aspirin 150 mg was given for pre-eclampsia prophylaxis starting before gestational week 16 and carried through to gestational week 36.

### 2.2. Data Collection

All patients were identified from a prospectively maintained clinic database [4]. Electronic patient records were reviewed to collect data on patient demographics, IBD characteristics, antenatal risk assessments, aspirin prescriptions, and antenatal/postnatal VTE prophylaxis. IBD characteristics included subtype of IBD, timing of diagnosis, clinical disease activity scores (physician’s global assessment [PGA] for each trimester ranging from 0 to 3, scored by a single clinician [CPS for all cases]), treatment with steroids, and IBD-related hospital admissions. Due to updates in the obstetric electronic patient records, risk assessments for VTE and pre-eclampsia were available only for women who attended from 2018 onwards.

### 2.3. Outcome Variables

Primary outcomes were the incidence of VTE, the incidence of pre-eclampsia, and disease flare whilst taking aspirin. Pre-eclampsia was defined as the development of hypertension (≥140/90 mmHg) accompanied by significant proteinuria or evidence of maternal organ dysfunction after 20 weeks gestation [8]. A disease flare was defined as the presence of at least one of the following: clinical disease activity score (PGA ≥ 1), treatment with steroids, and IBD-related hospital admission during pregnancy. Secondary outcomes were the prevalence of VTE prophylaxis and VTE risk classification.

### 2.4. Statistical Analysis

Data analysis was performed using Microsoft Excel (Version 2508) and GraphPad Prism (http://www.graphpad.com/quickcalcs/ accessed 1 August 2025). Categorical variables were recorded as counts and percentages, while continuous variables were summarised using means with standard deviation. Contingency tables were constructed, and the data were analysed using Chi-square test. A two-sided *p*-value of less than 0.05 was considered statistically significant.

### 2.5. Ethical Statement

The study was performed as a clinical service evaluation and audit, which are exempt from the requirement for ethics committee approval and individual patient consent [18].

## 3. Results

**Figure 1:**
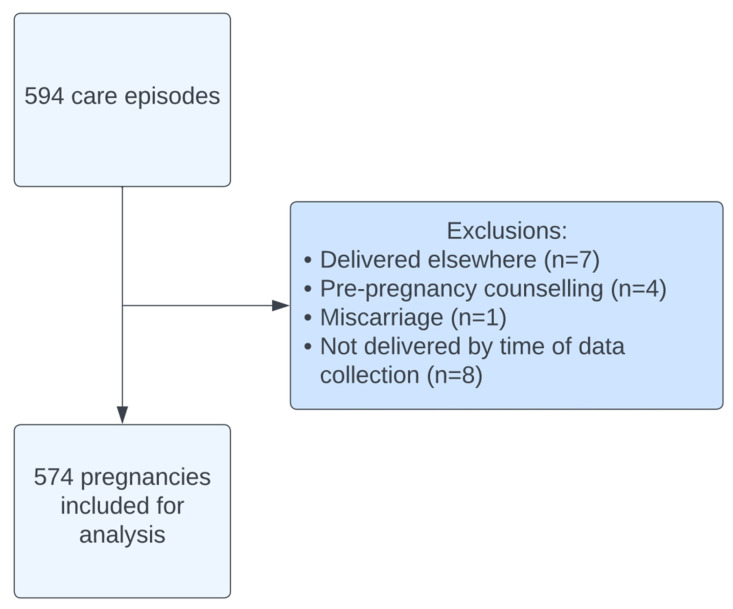
Cohort flow diagram.

A total of 594 care episodes were recorded between 2015 and 2024. After exclusions, 574 pregnancies (Figure 1) were included in this analysis. The average age was 31.9 ± 4.94 (mean ± SD) with an average body mass index (BM) of 26.8 ± 5.49. Ulcerative colitis accounted for approximately half (49.3%) of the diagnoses with 45.6% of women diagnosed with Crohn’s disease (Table 1). The majority (96.9%) of women were diagnosed with IBD prior to pregnancy. A total of 129 women experienced active disease during their pregnancy, the majority of which was classified as mild (55.0%) or moderate (38.8%).

### 3.1. VTE During Pregnancy

Over half (54.2%) of women were classified as low-risk for VTE antenatally, compared to 39.2% in the post-natal period (Table 2). Only 4.2% of women received antenatal VTE prophylaxis compared to 53.9% of postnatal women. Patients classed high-risk for VTE (9 of 13, 69.2%) were significantly more often prescribed VTE prophylaxis antenatally than those classed as low- or intermediate-risk (9 of 309, 2.9%; *p* < 0.00001; Supplementary Table S1).

The most common reasons for being classed high-risk for VTE antenatally were age (>35 years), high BMI (≥35 kg/m^2^) and hospital admission during pregnancy. Postnatally, 100% of high-risk patients were given VTE prophylaxis compared to 86% of low- or intermediate-risk patients (*p* < 0.00001; Supplementary Table S2). Postnatally the most common reasons for high-risk VTE classification were delivery factors, age (>35 years), parity (≥3) and obesity (BMI of ≥35 kg/m^2^). The overall incidence of VTE was low with only one case diagnosed during this period.

### 3.2. VTE in Patients with a Flare During the Third Trimester

A total of 51 women experienced a disease flare (PGA ≥ 2) during the third trimester (Supplementary Table S3), of which only 2 women received VTE prophylaxis. Both women receiving VTE prophylaxis were commenced during the first trimester for additional risk factors including previous VTE, family history, smoking and obesity. There was no statistically significant difference in the prevalence of VTE prophylaxis between disease flare and no disease flare (*p* = 0.880).

### 3.3. Aspirin and Pre-Eclampsia

Pre-eclampsia occurred in 15 cases (2.7%). Three cases developed severe pre-eclampsia requiring early delivery (<36 weeks). Indications for early delivery included Haemolysis, Elevated Liver enzymes and Low Platelets (HELLP) syndrome (n = 2) and eclampsia (n = 1). Two cases developed late-onset complications, one with HELLP syndrome with stillbirth at 37 weeks and one with eclampsia at 39 weeks. The majority of women (79.3%) were classified as low-risk for developing pre-eclampsia (Supplementary Table S4). Risk assessments were not available for three cases of severe pre-eclampsia with the remaining two cases classified as low-risk. A total of 67 women received aspirin prophylaxis throughout their pregnancy, most (n = 36, Supplementary Table S5) of which were high-risk. Of the 25 women classified as high-risk who did not receive aspirin, 10 were classified as high-risk due to a diagnosis of IBD alone with a further nine determined as high-risk due to a combination of IBD and first pregnancy. These cases were reviewed by a consultant obstetrician and deemed not to require aspirin prophylaxis. No statistically significant difference (Supplementary Table S6; *p* = 0.843) was identified in the incidence of pre-eclampsia across the aspirin and no-aspirin groups on non-adjusted analysis. When restricting the analysis in high-risk patients for pre-eclampsia there was no statistically significant (*p* = 0.354, Supplementary Table S7) difference in pre-eclampsia diagnosis between aspirin users and non-users. All three cases of severe pre-eclampsia requiring early delivery occurred in non-aspirin users.

### 3.4. Aspirin and Disease Flare

Most (n = 51; Table 3 and Supplementary Tables S8 and S9) women receiving aspirin prophylaxis remained in remission throughout their pregnancy. The risk of symptomatic disease flare was 21.6% for aspirin users versus 22.2% for non-users (*p* = 0.61). A total of 70 women required treatment with steroids, only 12 of which were taking aspirin prophylaxis. When restricting disease activity assessment to the harder endpoints of steroid requirement (*p* = 0.141) or admission during pregnancy (*p* = 0.882) no statistically significant differences were found (*p* = 0.141 and *p* = 0.882 respectively).

## 4. Discussion

Managing IBD during pregnancy requires excellent management of IBD itself and an equal focus of direct effects of IBD on obstetric aspects of care in the antenatal and post-partum period. The prevention of common complications of pregnancy such as VTE or pre-eclampsia also forms a vital part of holistic care. This study assessed the incidence of VTE, pre-eclampsia, and risk of disease flare from low-dose aspirin amongst pregnant women with IBD. We found a low risk of VTE and pre-eclampsia in this cohort. Importantly we also showed that the use of aspirin 150 mg daily as pre-eclampsia prophylaxis was not associated with an increased risk of IBD flares in this cohort.

Within our cohort, only one case of VTE was diagnosed, resulting in an incidence rate of 1.8 cases per 1000 pregnancies. This is in line with the overall incidence of VTE in pregnancy and the puerperium of 1–2 per 1000 women [7], suggesting no increased risk was seen in our study. While multiple studies have demonstrated an increased risk of pregnancy-associated VTE in women with IBD with adjusted relative risk increases ranging from 1.67 to 3.78 [9,19,20], the absolute risk remains modest [9]. Notably, the prevalence of VTE prophylaxis is not known within these patient cohorts. The majority of high-risk women, including all postnatal women, received VTE prophylaxis within this study, which may have reduced the overall incidence. As expected, the proportion of women classified as high-risk was greater in the postnatal period due to the accumulation of risk factors [7]. As a result of rigorous application of VTE risk assessments during the delivery period most high-risk patients received prophylaxis as suggested by guidelines.

In contrast, antenatal prophylaxis was not as rigorously applied, probably owing to variance of clinical practice and less focus of VTE risk assessment during clinical encounters. Only 2 of 51 patients with a symptom-based flare received VTE prophylaxis during the third trimester. This highlights an urgent need for improved assessment and prophylaxis. Previous studies have demonstrated an increased risk of pregnancy-associated VTE during disease flare [9,19]. Moreover, a systematic review with meta-analysis demonstrated a trend towards increased VTE risk, although this did not reach statistical significance [RR 7.81, 95% CI: 0.90–67.78] [10]. Importantly, the analysis identified the third trimester as the highest risk of thromboembolism during the antenatal period. Considering the combination of these factors, we are implementing measures to ensure VTE prophylaxis for all patients flaring during the third trimester by placing an increased focus during clinic and IBD helpline encounters. A total of 51 women experienced a moderate to severe disease flare during the third trimester within this cohort, none of whom were newly commenced on VTE prophylaxis. Nevertheless, no harm from VTE within the study period was found. While we may have overestimated the effects of active IBD by using a symptom rather than biomarker definition of active disease, we have nevertheless detected an area where improvement is needed. Given that outside pregnancy VTE prophylaxis is not usually required, this is an aspect that is potentially not often present in clinicians’ minds when treating pregnant patients with IBD. When potential disease flares during pregnancy are detected via IBD helplines, general IBD clinics, and in the setting of combined IBD antenatal clinics, the need for VTE prophylaxis should be considered for all women in the third trimester of pregnancy. Other IBD services, especially those without close working relationships with obstetric units, should audit VTE prophylaxis in their cohorts as this is a likely area of misses even in expert hands.

In our cohort the incidence of pre-eclampsia was low at 2.7%, compared to a pooled global prevalence of 4.6% (95% CI, 2.7–8.2%) [21]. Whilst autoimmune diseases such as systemic lupus erythematosus have been identified as a risk factor for the development of pre-eclampsia, such an effect has not been consistently demonstrated with IBD [2,11,22]. Whilst no increase in the overall rate of pre-eclampsia has been observed, a cohort study of over 85,000 births identified an increased risk of severe pre-eclampsia in women with IBD (HR 2.24, 95% CI 1.05–4.80) [11]. This effect was primarily driven by women receiving oral corticosteroids (HR 17.4, 95% CI 3.72–81.4) [11], implying severe IBD may predispose women to a more aggressive course of pre-eclampsia. Our findings are in line with the previous evidence that found no overall increased risk of pre-eclampsia for patients with IBD. A total of five women experienced severe complications of pre-eclampsia within this cohort. Unfortunately, complications of pre-eclampsia can occasionally occur amongst low-risk women. Given the overall low incidence of severe pre-eclampsia, we were not able to assess a potential association of IBD with severe pre-eclampsia further within our study.

Low-dose aspirin reduces the frequency of pre-eclampsia and its associated consequences [23]. We found no evidence that low-dose aspirin precipitates a disease flare in our cohort of women with IBD. This confirms the results of two previous studies that found no increase in clinical disease activity [24,25] or inflammatory biomarkers [24] in women receiving low-dose aspirin. European guidelines do not recommend low-dose aspirin use for all women with IBD [1]. Despite limited evidence, current global consensus guidelines recommend the use of low-dose aspirin in women with IBD by 12–16 weeks of gestation [12]. In the authors’ view, the evidence provides limited rationale for prescribing aspirin to all pregnant patients with IBD though the risk for flares or other harms appears low. Notably, within this cohort, most women were appropriately prescribed low-dose aspirin according to NICE guidelines [8], suggesting that concerns regarding disease flare did not act as a barrier to appropriate treatment. We found no statistically significant difference in the rates of pre-eclampsia between the aspirin and non-aspirin groups; however, this is likely due to the small number of cases of pre-eclampsia within this cohort. Our study was not powered to assess any protective effect of aspirin against pre-eclampsia, especially as this effect has already between demonstrated in large-scale randomised controlled clinical trials [23].

The main strength of this study is the single-centre cohort design and complete follow-up of pregnancy outcomes. This reduces variability in practice by ensuring a consistent approach to IBD care and obstetric risk assessment, as well as subsequent management. Nevertheless, this study is not without limitations. Recording of aspirin use within patient records was non-standardised, possibly resulting in missing cases. However, this number is likely minimal since a more in-depth review of records was conducted for women deemed intermediate- or high-risk. Moreover, our sample size was relatively small for rarer outcomes. This means the study is not powered to investigate associations between aspirin and non-aspirin groups. As inflammatory biomarkers and endoscopy were not routinely performed, we relied on subjective clinical disease activity scores to determine disease flares. PGA scoring is subjective and introduces a risk of bias. To overcome this, all PGA assessments were conducted by the same clinician throughout the study period. We focused on flares with significant clinical activity only and hence excluded PGA 1 from the flare analysis as variation in symptoms is common during pregnancy cases, even in remission. This may have introduced variability in disease severity classification. Finally, the results of our study may not be generalised to alternative centres due to variations in clinical practice. Naturally our study was not powered to assess the incidence of rare complications of pregnancy with precise certainty. When interpreting associations between aspirin exposure and outcomes in unadjusted comparisons, causality cannot be inferred.

In conclusion, our study we did not observe an increased risk of VTE or pre-eclampsia amongst our cohort of women with IBD. It is possible that our reduced incidence of VTE is secondary to relatively high coverage of VTE prophylaxis amongst high-risk women, particularly in the post-natal period. Moreover, we found no evidence that low-dose aspirin precipitates disease flare during pregnancy. Our findings are in consensus with growing evidence that low-dose aspirin is a safe treatment for the prevention of pre-eclampsia for women with IBD.

## Figures and Tables

**Table 1:** Patient demographics and IBD history. Values n (%).

Patient Demographics	Value
Age	Mean 31.9 ± 4.94
BMI	Mean 26.8 ± 5.49
**Smoking status**	
Never smoked	286 (73.7)
Smoked prior to conception	24 (6.2)
Current smoker	78 (20.1)
**IBD sub-type**	
Crohn’s disease	262 (45.6)
Ulcerative colitis	283 (49.3)
IBD unclassified	29 (5.0)
**Timing of diagnosis**	
Pre-existing diagnosis	556 (96.9)
New diagnosis during pregnancy	18 (3.1)
**Disease activity during pregnancy**	
Remission	445 (77.5)
Mild	71 (12.4)
Moderate	50 (8.7)
Severe	8 (1.4)

**Table 2:** Frequency table for VTE prophylaxis, risk and incidence of VTE.

Variable	Percentage of Women (n)
**VTE prophylaxis during**	
Yes	4.2 (22)
No	95.8 (500)
**VTE prophylaxis after**	
Yes	53.9 (280)
No	46.1 (240)
**Antenatal VTE risk**	
Low	54.2 (176)
Intermediate	41.8 (136)
High	4.0 (13)
**Postnatal VTE risk**	
Low	39.2 (135)
Intermediate	45.1 (155)
High	15.7 (54)
**VTE**	
Yes	0.18 (1)
No	99.8 (560)

**Table 3:** Association between Aspirin use during pregnancy and IBD disease activity = 0.610.

Disease Activity During Pregnancy	Aspirin During Pregnancy		Total
	Yes	No	
Remission	51	383	434
Mild	8	61	69
Moderate	6	43	49
Severe	2	6	8
Total	65	495	560

## Data Availability

Summary data are available on request.

## Supplementary Materials

The following supporting information can be downloaded at: https://www.mdpi.com/article/10.3390/jcm14228072/s1, Table S1: Contingency table for prevalence of antenatal VTE prophylaxis and antenatal VTE risk. (Chi-square 106.3573; *p* < 0.00001). Table S2: Contingency table for prevalence of postnatal VTE prophylaxis and postnatal VTE risk. (Chi-square 213.1665; *p* < 0.00001). Table S3: Contingency table for prevalence of VTE prophylaxis for patients with disease flare (PGA ≥ 2). (Chi squared 0.023; *p* = 0.880). Table S4: Frequency table for incidence of pre-eclampsia, antenatal pre-eclampsia risk and aspirin use during pregnancy. Table S5: Aspirin use depending on pre-eclampsia risk. (Chi-square 85.3609; *p* < 0.00001). Table S6: Contingency table, Chi-square 0.039 *p* = 0.843. Table S7: Contingency table for incidence of pre-eclampsia and aspirin use during pregnancy for high risk women. Chi-square 0.860, *p* = 0.354. Table S8; Contingency table for risk of flare that required steroid treatment during pregnancy between aspirin and non-aspirin users. Chi-square 2.168, *p* = 0.141. Table S9; Contingency table for risk of hospital admission for a flare that required steroid treatment during pregnancy between aspirin and non-aspirin users. Chi-square 0.022, *p* = 0.882 [26].
