# Supplementary material for: Low Incidence of Venous Thromboembolism and Pre-Eclampsia in Women Cared for in a Specialist Antenatal Clinic for Inflammatory Bowel Disease
Source: J Clin Med. 2025 Nov 14;14(22):8072. doi: 10.3390/jcm14228072 (PMC12653284; doi:10.3390/jcm14228072)
Supplement: Supplementary file 1 [file jcm-14-08072-s001.zip › jcm-3982817-supplementary.pdf]

Supplementary Material

Table S1: Contingency table for prevalence of antenatal VTE prophylaxis and antenatal VTE risk. (Chi-square 106.3573; $p < 0.00001$.)

Antenatal VTE risk	Antenatal VTE prophylaxis		Total
	Yes	No	
**Low**	2	173	175
**Intermediate**	7	127	134
**High**	9	4	13
	18	304	322

Table S2: Contingency table for prevalence of postnatal VTE prophylaxis and postnatal VTE risk. (Chi-square 213.1665; $p < 0.00001$).

Postnatal VTE risk	Postnatal VTE prophylaxis		Total
	Yes	No	
**Low**	6	129	135
**Intermediate**	128	26	154
**High**	54	0	54
	188	155	343

Table S3: Contingency table for prevalence of VTE prophylaxis for patients with disease flare (PGA ≥2). (Chi squared 0.023; p 0.880).

Disease flare in the third trimester	VTE prophylaxis prescribed		Total
	Yes	No	
Yes	2	49	51
No	19	415	434
Total	21	464	485

Table S4: Frequency table for incidence of pre-eclampsia, antenatal pre-eclampsia risk and aspirin use during pregnancy.

Variable	Percentage of women (n)
**Pre-eclampsia**	
Yes	2.7 (15)
No	97.3 (545)
**Aspirin during pregnancy**	
Yes	11.9 (67)
No	88.1 (494)
**Risk of pre-eclampsia**	
Low	79.3 (242)
Intermediate	0.7 (2)
High	20.0 (61)

Table S5: Aspirin use depending on pre-eclampsia risk. (Chi-square 85.3609; p < 0.00001)

**Pre-eclampsia risk**	**Aspirin during pregnancy**		**Total**
	**Yes**	**No**	
**Low**	19	222	241
**Intermediate**	0	2	2
**High**	36	25	61
**Total**	55	249	304

Table S6: Contingency table, Chi-square 0.039 P = 0.843

**Pre-eclampsia**	**Aspirin during pregnancy**		**Total**
	**Yes**	**No**	
**Yes**	2	13	15
**No**	63	477	540
**Total**	65	490	555

Table S7: Contingency table for incidence of pre-eclampsia and aspirin use during pregnancy for high risk women. Chi-square 0.860, P=0.354

**Pre-eclampsia**	**Aspirin during pregnancy**		**Total**
	**Yes**	**No**	
**Yes**	1	2	3
**No**	35	23	58
**Total**	36	25	61

Table S8; Contingency table for risk of flare that required steroid treatment during pregnancy between aspirin and non-aspirin users. Chi-square 2.168, P=0.141

**Steroid during pregnancy**	**Aspirin during pregnancy**		**Total**
	**Yes**	**No**	
**Yes**	12	58	70
**No**	54	434	488
**Total**	66	492	558

Table S9; Contingency table for risk of hospital admission for a flare that required steroid treatment during pregnancy between aspirin and non-aspirin users. Chi-square 0.022, P=0.882

**Admission during pregnancy**	**Aspirin during pregnancy**		**Total**
	Yes	No	
**Yes**	7	55	62
**No**	51	427	478
**Total**	57	483	540
